# Correction: [Pt(*O,O'*-acac)(γ-acac)(DMS)] Alters SH-SY5Y Cell Migration and Invasion by the Inhibition of Na^+^/H^+^ Exchanger Isoform 1 Occurring through a PKC-ε/ERK/mTOR Pathway

**DOI:** 10.1371/journal.pone.0118490

**Published:** 2015-03-06

**Authors:** The PLOS ONE Staff

In the fourth sentence of the second paragraph under the subheading “[Pt(acac)_2_(DMS)] decreases expression and activity of MMP-2 and -9” in the Results section, the units for SB203580 should be listed in μM instead of mM.

The correct sentence is: When SH-SY5Y cells were pretreated for 30 min with SB203580 (1 and 10 μM), and then with 0.50 μM [Pt(acac)_2_(DMS)] for 24 h, the inhibition of MMP-2 and MMP-9 expression levels was blocked (Fig. 4D), but the effects of [Pt(acac)_2_(DMS)] on Matrigel invasion were unchanged (Fig. 4E).

## References

[1] Muscella A, Vetrugno C, Calabriso N, Cossa LG, De Pascali SA, Fanizzi FP, et al (2014) [Pt(*O,O’*-acac)(γ-acac)(DMS)] Alters SH-SY5Y Cell Migration and Invasion by the Inhibition of Na^+^/H^+^ Exchanger Isoform 1 Occurring through a PKC-ε/ERK/mTOR Pathway. PLoS ONE 9(11): e112186 doi:10.1371/journal.pone.0112186 25372487 10.1371/journal.pone.0112186 PMC4221608

